# Volatilomics-Based Discovery of Key Volatiles Affecting Flavor Quality in Tomato

**DOI:** 10.3390/foods13060879

**Published:** 2024-03-14

**Authors:** Zhonghui Zhang, Weizhen Ye, Chun Li, Haihong Zhou, Chao Wang, Penghui Liu, Binxin Zhou, Hanqing Zhao, Shouchuang Wang, Jun Yang

**Affiliations:** 1 School of Breeding and Multiplication (Sanya Institute of Breeding and Multiplication), Hainan University, Sanya 572025, China; zhonghui.zhang@hainanu.edu.cn (Z.Z.); weizhen.ye@hainanu.edu.cn (W.Y.); chun.li@hainanu.edu.cn (C.L.); haihong.zhou@hainanu.edu.cn (H.Z.); chaowang@hainanu.edu.cn (C.W.); penghui.liu@hainanu.edu.cn (P.L.); shouchuang.wang@hainanu.edu.cn (S.W.); 2 School of Tropical Agriculture and Forestry, Hainan University, Haikou 570228, China; 3 College of Traditional Chinese Medicine, Hebei University, Baoding 071000, Chinazhaohq@hbu.edu.cn (H.Z.)

**Keywords:** tomato, volatiles, sensory evaluation, HP-SPME/GC-MS

## Abstract

Volatile accumulation during tomato ripening greatly affects the fruit flavor. In this study, four accessions from each of the three tomato subgroups (BIG, *S. lycopersicum*, CER, *S. lycopersicumvar. Cerasiforme*, and PIM, *S. pimpinellifolium*) were subjected to a sensory evaluation. The CER subgroup had the highest fruit-flavor score. Using a Headspace solid-phase microextraction/gas chromatography-mass spectrometer (HP-SPME/GC-MS), a volatile database containing 94 volatiles was created. Pentanal accumulated in green fruits and 1-pentanol in red fruits. 1-Octen-3-ol was discovered to underlie the bitterness of green tomatoes, and it was most abundant in PIM green fruits. Phenylethyl alcohol affected the acidity and sweetness of red tomatoes, and it was most abundant in CER red fruits. Branched-chain volatiles were most abundant in PIM and BIG red fruits, while apocarotenoids were most abundant in CER red fruits. These findings suggest that domestication and improvement have influenced volatile content, and apocarotenoids and branched-chain volatiles synergistically mediated aromatic flavors in red fruits. This study provides a metabolic basis for analyses of the molecular mechanisms of fruit-flavor formation.

## 1. Introduction

Tomato is one of the most important vegetable crops worldwide and is loved by consumers because of its unique flavor [1]. Flavor, as an important nutritional quality of tomatoes, results from the interaction of sugars, acids, and organic volatiles within tomato fruits [2]. More than 400 organic volatiles have been detected in mature tomato fruits, but only a few significantly affect flavor quality [3,4]. Tomato organic volatiles can be divided into four categories: branched-chain amino acid derivatives, carotenoid derivatives, fatty acid derivatives, and phenylpropane derivatives [5]. The following key volatiles affect the nutritional quality of tomato fruits and have been associated with particular aromas favored by consumers: 2-phenylacetaldehyde (floral), 3-methylbutanol (malt), 6-methyl-5-hepten-2-one (fruity and floral), and hexanal (grassy). A number of other volatiles are known to underlie aromatic, sour, and sweet flavors in tomato fruits [6,7]. Organic volatiles account for 62% of the sweet and 22% of the sour flavors of tomato fruits [8].

Tomato is a climacteric fruit in which the accumulation of metabolites varies significantly during ripening [9]. The 2-isobutylthiazole, 6-methyl-5-hepten-2-one, *cis*-3-hexenal, geranylacetone, hexanal acetone, and *trans*-2-hexenal continued to increase during tomato-fruit ripening [10]. At maturity, 2-methylbutanal, 2-methylbutanol, 2-phenylacetaldehyde, 3-methylbutanal, 3-methylbutanol, 6-methyl-5-hepten-2-ol, and 6-methyl-5-hepten-2-one are abundant in tomato fruits [11]. There are differences in the composition of volatiles between green and red fruit, and most volatiles accumulate in red fruit [12]. At immature stages, most tomato-fruit volatiles exist in glycosylated forms that are then released when the fruit is ripe. For example, glycosylated salicylic acid accumulates in immature tomato fruits and is released as free salicylic acid as the fruit ripens [13].

Tomato-fruit flavor is affected by many factors such as genetics, growth environment, cultivation method, and storage conditions [2,11,13,14,15]. Significant differences in volatile accumulation have been documented during domestication between CER and PIM fruits, with CER varieties having a superior flavor quality [16]. Due to yield, nutrition and resistance have been focused on, leading to a decline in the flavor quality of tomato fruits [17]. To improve fruit-flavor attributes, high-flavor-quality parental plants should be selected to breed new tomato varieties with desirable flavor qualities. In terms of flavor’s molecular biology, key genes affecting tomato-fruit flavor have been discovered, and the molecular regulatory network underlying volatile accumulation is characterized as part of efforts to improve the flavor qualities of contemporary tomato cultivars [18,19,20].

At present, studies of tomato-fruit flavor largely fall into two categories, representing either detailed investigations of volatile accumulation in select tomato materials or broad-scale surveys of natural variation among tomato populations in volatile accumulation during fruit ripening. To date, relatively few studies have examined the relationship between volatile accumulation and flavor quality during fruit ripening across tomato subgroups. In this study, four accessions from each of the BIG (*S. lycopersicum*), CER (*S. lycopersicumvar. cerasiforme*), and PIM (*S. pimpinellifolium*) tomato subgroups were selected to investigate how volatile accumulation during ripening affects flavor quality. A sensory evaluation was performed to characterize flavor changes during fruit ripening, and a metabolic database containing 94 volatiles was constructed for tomatoes using HP-SPME/GC-MS. 1-Octen-3-ol was discovered to underlie the bitterness of green tomatoes, and it was most abundant in PIM green fruits. Phenylethyl alcohol affected the acidity and sweetness of red tomatoes, and it was most abundant in CER red fruits. Branched-chain volatiles were most abundant in PIM and BIG red fruits, while apocarotenoids were most abundant in CER red fruits. These findings suggest that domestication and improvement have influenced volatile content, and apocarotenoids and branched-chain volatiles synergistically mediated aromatic flavors in red fruits. This study provides data to support in-depth analyses of flavor differences among tomato subgroups and theoretical support for parental selection in tomato-flavor breeding.

## 2. Material and Methods

### 2.1. Plant Materials and Sampling

In this study, green and red fruits from three tomato subgroups were used to study the tomato volatilomics. Four varieties were selected from each of the three subgroups, for a total of twelve varieties. Tomato materials were collected from the Agricultural Science Experimental Base of Hainan University, in Danzhou, China. Tomato fruits 35 DPA (days post-anthesis) were selected as green-fruit material, and tomato fruits 55 DPA (days post-anthesis) were selected as red-fruit material [21]. Fruits with uniform growth and a similar size, hardness, and color were selected as research materials. Three plants constitute one biological replicate, with a total of three biological replicates. Fruits were sampled from each plant at 8 am and these were collected in 50 mL polypropylene centrifuge tubes and then placed in liquid nitrogen.

### 2.2. Reagents and Standards

EDTA was procured from the Sinopharm Chemical Reagent Company. Additional chemicals were purchased from the Shanghai Aladdin Biochemical Technology Co., Ltd. (Aladdin, Shanghai, China), including the following: analytical grade ethanol and calcium chloride dehydrate; an alkane standard (C6-C20) used to calculate the retention index (RI); twelve volatile standards (i.e., 1-nitro-pentane, 1-octen-3-ol, 2-isobutylthiazole, acetophenone, *α*-terpineol, *β*-ocimene, citral, geranyl acetone, methional, neral, *trans*-*β*-Ionone, and undecane); and ethyl nonanoate as the internal target [22].

### 2.3. Sensory Analysis

A quantitative descriptive analysis (QDA) was carried out by a trained panel of six college students (three women and three men, aged 25–30), who signed a consent form. They evaluated and rated the flavor of tomato fruits belonging to all three subgroups and both maturity stages. The tomato fruits were cut into equally sized pieces, numbered, and randomly assigned to each group member. Panel members did not interact with each other during the evaluation. Between samples, panel members took a 30 s break. A rating scale was devised to reflect how much the panel members enjoyed the tomato samples; scores ranged from zero to six. After testing, scores were averaged across the six panelists; these mean scores were used as the final score for each sample [22]. The average score for each flavor indicator for each tomato variety was also calculated. The ratio of the average scores (for the flavor indicators) between the two subgroups was used as an indicator of subgroup differences. The study was approved by the Research Ethics Committee of the Affiliated Hospital of Hebei University (File number: HDFYLL-KY-2023-084).

### 2.4. Sample Preparation and Extraction

Frozen tomato-fruit samples were freeze-dried (ALPHA 2-4 LD plus, Christ, Osterode, Germany) and then ground using a mixer mill (MM 400, Retsch, Haan, Germany) with a zirconia bead for 30 s at 30 Hz. For each sample, 0.5 g of the resulting powder was transferred into a 22 mL glass bottle, and then calcium chloride dihydrate (1 g), EDTA-NaOH (1 mL, 100 mM), and ethyl nonanoate ethanol solution (10 µL, 100 ppm) were added in sequence. The mixture was vortexed thoroughly. Samples were pre-heated at 50 °C for 10 min and then extracted for 20 min at 50 °C prior to GC-MS analysis [23].

### 2.5. HS-SPME/GC–MS Analysis

Gas chromatography (7890B GC, Agilent Technologies, Santa Clara, CA, USA) combined with a triple quadrupole mass spectrometer (7000D MS, Agilent Technologies, Santa Clara, CA, USA) was utilized to detect volatile compounds. The capillary column used to separate volatile compounds was an HP-5 MS (30 m × 0.25 mm i.d. × 0.25 μm film thickness; Agilent Technologies, Santa Clara, CA, USA). To ensure an adequate separation of volatiles, the GC oven temperature program was set to 40 °C (3 min) initially, ramping to 160 °C at 2 °C/min, and then to a final temperature of 300 °C (3 min) with a temperature increase of 50 °C/min. The gasification-chamber temperature was 270 °C in the non-shunt injection mode. Helium was used as the carrier gas (purity 99.999%) with a flow rate of 1.2 mL/min. The mass spectrum temperature was 270 °C. The ion source temperature was 300 °C. The scanning range of the full scan mode was 40–650 *m*/*z* [23].

### 2.6. Identification and Quantification of Volatiles of Tomato Fruits

Qualitative analysis of volatiles: A heating procedure was used to characterize mixed solutions of C8-C20 alkanes. MS-DIAL (version 4.8) was used to obtain the actual retention index (RI) of each volatile. Next, the detected signals were matched with metabolites in the NIST 17 spectrogram library. Matches were determined by Match and R Match values (≥850) and confirmed when the difference between metabolite RI and the RI obtained by MS-DIAL was ≤20. Using these criteria, metabolic signals were annotated. Standards were used to identify volatiles with low partial matching scores to ensure the accuracy of the qualitative results.

Quantitative analysis of volatiles: Feature fragments with high abundance in mass spectrometry were selected for semi-quantitative integration. MassHunter Quantitative Analysis was used to analyze the original data. Quantitative and qualitative ion peaks were aligned to determine if they had the same RT range and similar peak shape. These comparisons were used to manually adjust the peak area of the previous integration error. The internal standard method was used to normalize the peak area [22,23].

### 2.7. Metabolome Data Analysis

Data were compared using *t*-tests in Excel. Total ion chromatograms (TIC) were drawn using Origin 2022, while pie charts and bar charts were created using GraphPad Prism 8. Orthogonal partial least squares discriminant analysis (OPLS-DA) and principal component analysis (PCA) were performed using SIMCA14.1 [22,23]. Heat maps were created and cluster analysis was performed using Tbtools v1.108.

## 3. Results

### 3.1. Construction of a Tomato-Fruit Volatiles Database

**Figure 1:**
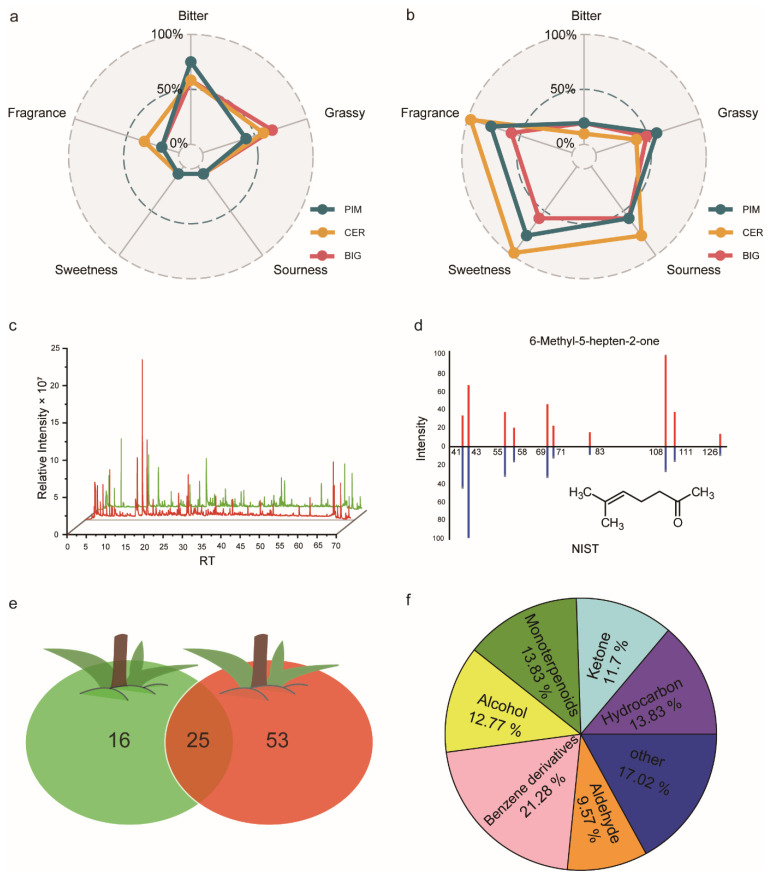
Construction of a metabolic database for tomato volatiles based on sensory evaluation. Radar map of sensory-evaluation data for the three tomato subgroups collected at (**a**) immature and (**b**) mature stages. Here, the tomato subgroups are identified by color: PIM (blue), CER (yellow), and BIG (red). (**c**) Total ion chromatogram (TIC) of tomato volatiles in immature (green) and mature (red) fruits. The green and red lines represent green and red tomato fruit respectively. (**d**) 6-Methyl-5-hepten-2-one was identified using the National Institute of Standards and Technology (NIST) database. The red and blue represent metabolite annotated signal and standard fragments respectively. (**e**) The number of volatiles detected in green and red fruits. The green and red represent green and red tomato fruit respectively. (**f**) Classification of volatiles that were detected in tomato.

To investigate flavor changes among tomato subgroups during ripening, both green and red fruits from all PIM, CER, and BIG accessions were included in the sensory evaluation. The sensory evaluation indexes included aromatic, bitter, grassy, sour, and sweet flavors. At the green (immature) fruit stage, PIM fruits had the most bitter flavor, while BIG fruits exhibited a more pronounced grassy flavor and CER fruits had a distinctive aromatic flavor (Figure 1a). At the red (mature) fruit stage, the acidic flavor of CER fruits was 1.33 times higher than that of BIG and PIM fruits; sweet and aromatic flavors were 1.67 times higher in CER fruits than in BIG fruits and 1.25 times higher than in PIM fruits (Figure 1b). There were differences between the flavor qualities of tomato fruits of different subgroups, with CER subgroup fruits having a higher acidity and sweetness and a higher aromatic flavor.

Mixed samples of green and red tomato fruits were analyzed using HP-SPME/GC-MS. A comparison of the total ion current (TIC) plots of green and red tomato fruits revealed that red tomato fruits had more volatile volatiles, with a higher number and abundance of signals before 35 min g in red fruits than in green fruits (Figure 1c and Supplementary Table S3). The results indicated significant differences in the composition of volatiles between green fruit and red fruit. After processing the raw metabolic data with MS-DIAL software (version 4.8) and matching it against the NIST database, we identified a total of 94 volatiles, such as 6-methyl-5-hepten-2-one (Figure 1d). Of these, 41 volatiles were detected in green fruits, and 78 volatiles were found in red fruits (Figure 1e). The volatiles identified included benzene derivatives (21.28%), terpenoids (13.83%), hydrocarbons (13.83%), alcohols (12.77%), ketones (11.70%), and aldehydes (9.57%) (Figure 1f).

### 3.2. Volatile Accumulation during Tomato-Fruit Ripening

**Figure 2:**
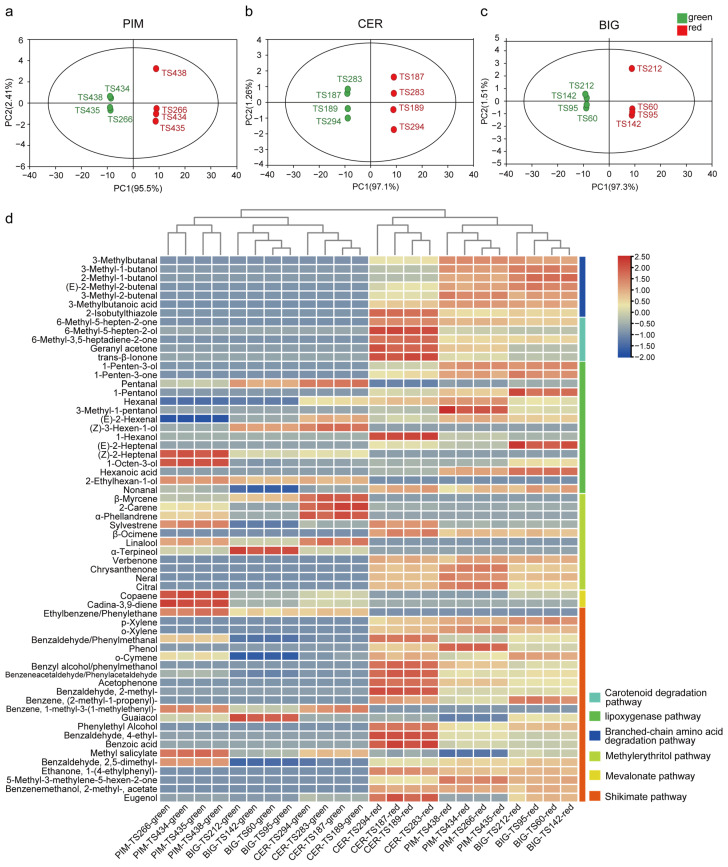
Differences in volatile accumulation between green and red tomatoes. (**a**–**c**) PCA analysis of PIM, CER, and BIG accessions. (**d**) Hierarchical cluster analysis of the three tomato subgroups. (Green indicates immature tomato samples, and red represents mature samples).

Principal components analysis was used to evaluate the volatile data for green and red fruits from the three tomato subgroups (BIG, CER, and PIM). The principal component analysis (PCA) results were similar for all three subgroups, with green and red fruits forming two distinct groups (Figure 2a–c). The results indicated that there were differences in the accumulation of volatiles between green fruit and red fruit. A hierarchical cluster analysis showed that volatiles related to the lipoxygenase (LOX) pathway, shikimate pathway, and methylerythritol pathway increased with the transition to red fruits. The pentanal accumulated at high levels in green fruits, while 1-penten-3-ol, 1-penten-3-one, 1-pentanol, and 3-methyl-1-pentanol were present at high levels in red fruits (Figure 2d). Apocarotenoid and branched-chain volatiles were also found in large amounts in red fruits. These results indicated that the variety of volatiles in tomatoes increased during the transition from green fruit to red fruit.

During fruit ripening, two volatiles markedly increased in abundance in green fruits: pentanal (higher in CER versus BIG and PIM) and z-3-hexen-1-ol (lower in PIM versus BIG and CER). In red fruits, several volatiles were identified at high abundance; these included 1-pentanol (higher in BIG versus PIM and CER) and 3-methyl-1-butanol (higher in PIM and BIG versus CER), as well as 1-hexanol and 6-methyl-5-heptene-2-one (both higher in CER versus PIM and BIG) (Figure 2d). The results indicated that the variation trend of volatiles in the fruit ripening of different subgroups was similar. The contents of volatiles in different subgroups may be affected by tomato domestication and improvement.

### 3.3. Relationship between Volatile Accumulation and Flavor Quality during Tomato Domestication and Improvement

**Figure 3:**
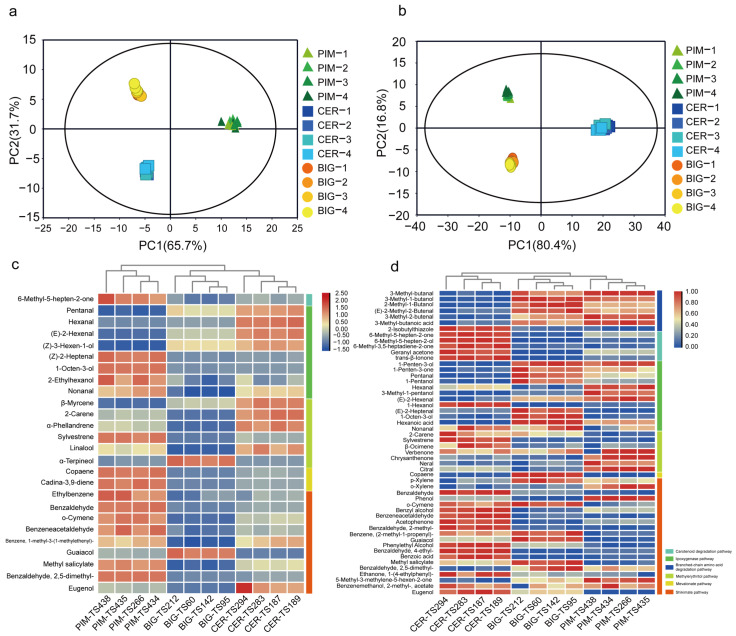
Differences in volatile accumulation among the three tomato subgroups (BIG, CER, and PIM). PCA of the three tomato subgroups for (**a**) immature and (**b**) mature fruit samples. Hierarchical cluster analysis of the three tomato subgroups for (**c**) immature and (**d**) mature fruits.

In this study, the three tomato subgroups formed distinct clusters in the PCAs for both green and red fruits (Figure 3a,b). For green fruits, a hierarchical cluster analysis revealed that 1-octen-3-ol and nonanal accumulated to the greatest extent in the PIM subgroup, while hexanal and pentanal were highest in the CER subgroup. Most of the methylerythritol pathway-related volatiles accumulated in large quantities in the CER subpopulation. In contrast, sylvestrene was highest in the PIM subgroup and α-terpineol in the BIG subgroup. Most of the volatiles related to the mevalonate and shikimic acid pathways accumulated in large quantities in the PIM subgroup, while guaiacol was highest in the BIG subgroup and eugenol in the CER subgroup (Figure 3c). These results indicate variations in the volatile compositions of green fruit tomatoes across different subgroups. In red fruits, a hierarchical cluster analysis revealed that branched-chain volatiles were most abundant in the PIM and BIG subgroups, while apocarotenoid was most abundant in the CER subgroup. Pentanal and 1-octen-3-ol were most common in BIG, (E)-2-hexenal in PIM, and 1-hexanol in CER. Most of the volatiles related to the shikimate pathway accumulated in large amounts in CER (Figure 3d). These results indicate differences in the accumulation of volatiles among different the subgroups of red fruit.

**Figure 4:**
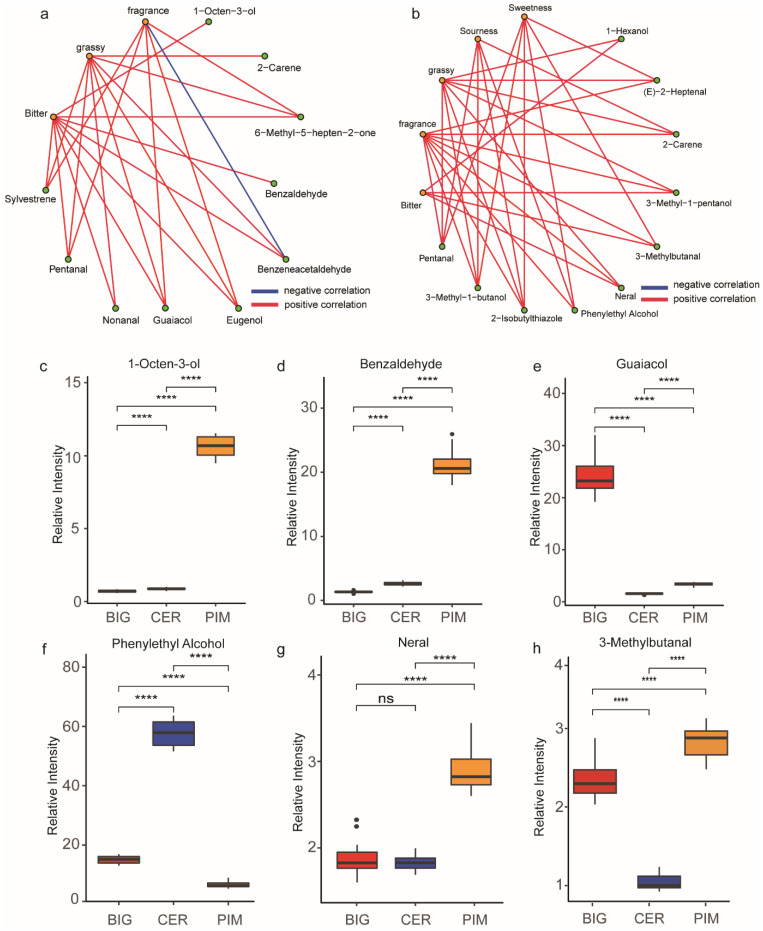
Statistical analysis of key volatiles in different tomato subgroups and at different stages of ripening. Correlation network for volatile abundance and sensory evaluations of (**a**) immature and (**b**) mature fruits. Key volatiles for immature fruits: (**c**) 1-octen-3-ol, (**d**) benzaldehyde, and (**e**) guaiacol. Key volatiles for mature fruits: (**f**) phenylethyl alcohol, (**g**) 3-methylbutanal, ns: not significant, and (**h**) neral. Results of Student’s *t*-test: ****, *p* < 0.001.

Based on the results of the correlation analysis between the sensory evaluation of green fruit and the quantitative data, a number of volatiles (1-octen-3-ol, 6-methyl-5-heptene-2-one, benzaldehyde, benzeneacetaldehyde, eugenol, guaiacol, nonanal, pentanal, and sylvestrene) were found to be highly correlated (*r* > 0.6) with aromatic, bitter, and grassy flavors (Figure 4a and Supplementary Table S7). Combining these findings with the flavor testing results from the FooDB website, it is deduced that 1-octen-3-ol, benzaldehyde, benzeneacetaldehyde, eugenol, and guaiacol may be the main compounds affecting the bitter taste of green fruit, while 2-carene, benzeneacetaldehyde, guaiacol, and nonanal may be the main compounds affecting grassy flavors in green fruits. The accumulation of 1-Octen-3-ol and Benzaldehyde in PIM, CER, and BIG subgroups was similar to the bitter scores for green fruits across subgroups (Figure 1a and Figure 4c,d). This suggests that 1-octen-3-ol and benzaldehyde are key volatiles contributing to bitterness in different subgroups of green fruits. Guaiacol was more abundant in BIG versus CER and PIM, which was similar to the grass taste scores for green fruits across subgroups (Figure 1a and Figure 4e). This suggests that guaiacol is the key volatile responsible for grassy flavors in the different subgroups of green fruits.

Based on the results of the correlation analysis between the sensory evaluation of red fruit and the quantitative volatile data, a number of volatiles (1-hexanol, 2-carene, 2-isobutylthiazole, 3-methyl-1-butanol, 3-methyl-1-pentanol, 3-methylbutanal, (E)-2-heptenal, neral, pentanal, and phenylethyl alcohol) were found to be correlated (*r* > 0.6) with aromatic, bitter, grassy, sour, and sweet flavors (Figure 4b and Supplementary Table S8). Combining these findings with the flavor testing results from the FooDB website, it is deduced that 3-methyl-1-butanol, 3-methylbutanal, and phenylethyl alcohol may be the main compounds affecting the aromatic flavors of red fruit, while 3-methyl-1-butanol and phenylethyl alcohol may be the main compounds affecting the sour and sweet flavors of red fruit, and 2-isobutylthiazole and 3-methylbutanal may be the main compounds affecting the bitterness of red fruit. Phenylethyl alcohol was significantly more abundant in CER than in BIG and PIM, and was similar to the sour and sweet scores for red fruits across subgroups (Figure 4f). The concentration of Neral in PIM is higher than in BIG and CER (Figure 4g), aligning with the scores for grassy flavors in red fruits (Figure 1b). This result suggests that Neral is a key volatile contributing to the grassy flavor. The concentration of 3-methylbutanal in PIM is higher than in BIG and CER, aligning with the bitter taste scores in red fruits (Figure 1b and Figure 4h). This result indicates that 3-methylbutanal is a key metabolite responsible for the bitterness in tomatoes.

### 3.4. Volatile Metabolic Pathways Affect Tomato Flavor Quality

An OPLS-DA on the quantitative volatile data identified separate clusters for green and red tomato fruits (Supplementary Figure S1). Through differential metabolite analysis, 16 differentially expressed metabolites with VIP values greater than one were identified (Supplementary Figure S2); these mainly included apocarotenoids, benzene volatiles, branched-chain volatiles, and terpenoid volatiles. A hierarchical cluster analysis revealed that most volatiles accumulated in large quantities in red fruits, with only a few volatiles accumulating in green fruits. 6-Methyl-5-heptene-2-one, phenylethyl alcohol, and *trans*-*β*-ionone accumulated only in red fruits, while guaiacol and methyl salicylate accumulated only in green fruits (Supplementary Figure S3), demonstrating differences in volatile composition between green and red tomatoes.

**Figure 5:**
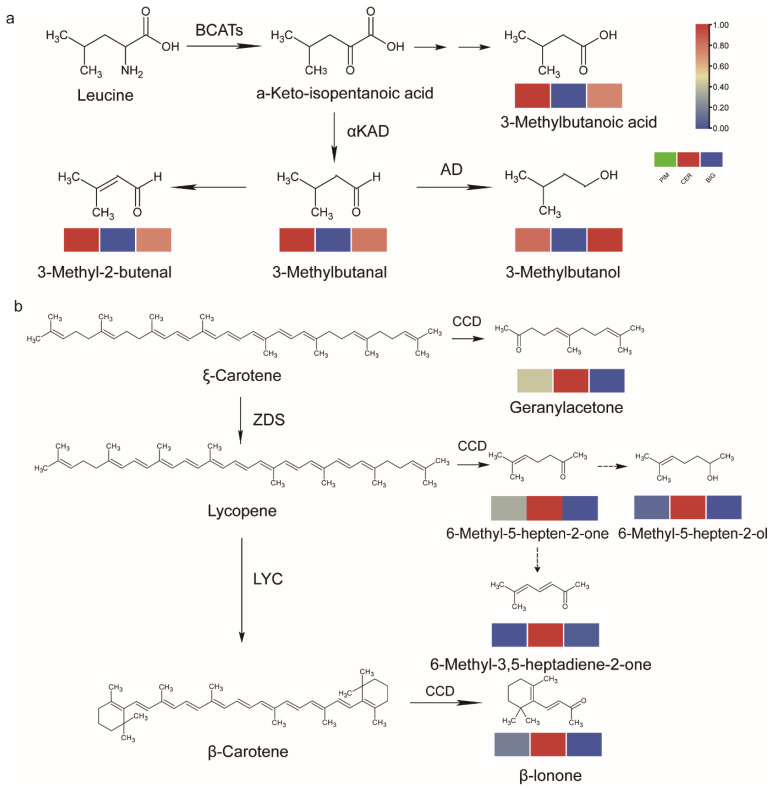
Biosynthetic pathways for key flavor volatiles: (**a**) branched-chain amino acid-degradation pathway and (**b**) carotenoid-degradation pathway. The heatmaps were drawn based on relative volatile contents, and the abundance of each metabolite was normalized. In the heatmap columns, the PIM, CER, and BIG subgroups are represented from left to right.

The correlation analysis of apocarotenoid and branched-chain volatiles in red fruits identified strong correlations between 3-methyl butanoic acid and 3-methyl-1-butanol, 3-methyl-2-butanal, and 3-methylbutanal. In addition, the correlation analysis of apocarotenoid in red fruits identified that 6-methyl-3,5-heptadiene-2-one and 6-methyl-5-hepten-2-ol was highly correlated with 6-methyl-5-heptene-2-one, as well as with geranyl acetate and *trans*-*β*-ionone (Supplementary Figure S4). 3-methyl-butanoic acid may complement degradation pathways for branched-chain amino acids, and 6-methyl-3,5-heptadiene-2-one and 6-methyl-5-hepten-2-ol are likely downstream products of 6-methyl-5-heptene-2-one (Figure 5).

Branched-chain volatiles accumulated in large amounts in PIM and BIG versus CER, while apocarotenoid accumulated in large amounts in CER versus PIM and BIG. 3-methylbutanal, 3-methyl-1-butanol, and 3-methyl-2-butenal have a fruity flavor, as well as 6-methyl-3,5-heptadiene-2-one, 6-methyl-5-hepten-2-ol, 6-methyl-5-heptene-2-one, Geranyl acetone, and *trans*-*β*-Ionone had aromatic flavors (Figure 5). This suggests that the aromatic flavor of red tomato fruits is determined by branched-chain volatiles and apocarotenoid. Branched-chain volatiles mainly affected the fruit aromatics of PIM and BIG. Apocarotenoid mainly affected the fruit aromatics of CER.

## 4. Discussion

In this study, apocarotenoid and branched-chain volatiles accumulated in large amounts in red tomato fruits. The branched-chain volatiles 3-methyl-1-butanol, 3-methyl-2-butenal, and 3-methylbutanal were present at high levels in the PIM and BIG subgroups, while the apocarotenoid volatiles 6-methyl-3,5-heptadiene-2-one, 6-methyl-5-hepten-2-ol, 6-methyl-5-heptene-2-one, geranyl acetone, and *trans*-*β*-ionone were most abundant in the CER subgroup (Figure 2d). Geranyl acetone and *trans*-*β*-ionone have been reported to be consistently elevated during tomato ripening [24], while 2-methylbutanal, 3-methyl-1-butanol, and 3-methylbutanal accumulate in large quantities at the breaker and ripening stages [25]. Comparing the CER and PIM subgroups, over the entire fruit lifespan, 6-methyl-5-hepten-2-ol was more abundant in CER than in PIM, and 3-methyl-1-butanol was more abundant in PIM than in CER [16]. The above results indicate the presence of differences in volatile accumulation among tomato subgroups.

In apple populations, differences in fruit volatile composition were found among accessions, with variation in the abundance of 23 volatiles linked to hereditary factors [26]. Genetically based differences in volatile composition were also found among tomato accessions in studies of various tomato cultivars [27,28]. In strawberry hybrid populations, differences in volatile content were found among strawberry fruits in the F1 generation [29]. In tomato-hybrid and introgression-line populations, differences in fruit volatile composition were also found among materials [30]. Based on the above results, differences in volatile composition among tomato subpopulations may have a genetic basis. Differences in volatile composition were found between geographically distinct rosemary (*Salvia rosmarinus* Schleid.) populations [31]. Similarly, differences in volatile composition were found between Campbell Early and Muscat of Alexandria grape varieties. Campbell Early grapes produce mainly C6 volatiles, lactones, and manganic acid derivatives, while Muscat of Alexandria grapes produce monoterpenes and sesquiterpenes [32]. Significant differences in volatile accumulation were also found among fir needles from populations with allopatric distributions [33]. In a recent study of tomato fruit volatiles, differences in volatile composition were found among geographically isolated tomato populations [34]. These findings suggest that differences among tomato subgroups in volatile compositions and flavor shifts during fruit ripening were mainly influenced by genetics and geography.

Organic volatiles play an important role in influencing not only fruit odor but also fruit taste. In this study, 1-octen-3-ol, benzaldehyde, benzeneacetaldehyde, eugenol, and guaiacol were found to be the key volatiles underlying the bitter flavor of green fruits; 3-methyl-1-butanol and phenylethyl alcohol were the primary volatiles affecting sourness and sweetness in red fruits. In other studies of blueberry and tomato, organic volatiles affected the sweetness and sourness of both tomato and blueberry fruits, but to different degrees [10,35]. Volatiles affecting sweetness included benzene-derived volatiles, carotenoid-derived volatiles, fatty acid-derived volatiles, and non-aromatic amino acid-derived volatiles [8]. These results suggest that organic volatiles affected the acidity and sweetness of tomato fruits.

In a study of strawberry-fruit sweetness, 2-pentanal, butanoic acid, ethyl ester, and hexanoic acid were found to play important roles in determining fruit sweetness [35,36]. Using FooDB to query volatile odor and taste [37,38], 2-pentanal and butanoic acid were linked to sweetness, indicating that volatile sweetness affects fruit taste. Similarly, 3-methyl-1-butanol and phenylethyl alcohol were also linked to sweetness in FooDB and may be key volatiles affecting sweetness in ripe tomatoes. A search through the FooDB website revealed that 1-octen-3-ol, benzaldehyde, and benzeneacetaldehyde are known to have a bad taste and that eugenol and guaiacol can underlie bitterness in fruit [39]. Thus, 1-octen-3-ol, benzaldehyde, benzeneacetaldehyde, eugenol, guaiacol, and nonanal are likely key metabolites underlying the bitter taste of green tomatoes.

Volatiles related to branched-chain amino acid and carotenoid-degradation pathways accumulate in large quantities in ripe (red) tomatoes [40]. In this study, 2-methylbutanal, 2-methylbutanol, 3-methylbutanal, 3-methylbutanol, 6-methyl-3,5-heptadiene-2-one, and 6-methyl-5-hepten-2-ol were identified specifically in red tomatoes. Branched-chain volatiles were most prevalent in PIM and BIG, while apocarotenoids were most common in CER. Organic volatiles such as 6-methyl-3,5-heptadiene-2-one, 6-methyl-5-hepten-2-ol, 6-methyl-5-heptene-2-one, geranyl acetone, and *trans*-*β*-ionone are uncommon in modern tomato varieties [41]. Heirloom tomato varieties also contain lower levels of branched-chain volatiles than cultivars [42]. These results suggest significant differences in the accumulation of branched-chain volatiles and deacylated carotenoids among the three subgroups (BIG, CER, and PIM). These two types of volatiles play important roles in influencing aromatic flavors in tomato fruits, with branched-chain volatiles having a fruity flavor and apocarotenoids having a floral flavor [43,44,45]. Differences in abundance between apocarotenoids and branched-chain volatiles may be responsible for variation in fruits’ aromatic flavors among the three tomato subgroups.

This study expands on previously reported synthesis pathways for apocarotenoids and branched-chain volatiles. Branched-chain volatiles such as 2-methylbutanal, 2-methylbutanol, 3-methylbutanal, and 3-methylbutanol are synthesized via branched-chain amino acid degradation [41,46]; 6-methyl-5-heptene-2-one, geranyl acetone, and *trans*-*β*-ionone are produced via lycopene degradation [41]. Here, 3-methylbutanoic acid was highly correlated with 3-methyl-1-butanol, 3-methyl-2-butenal, and 3-methylbutanal. As 3-methylbutanoic acid is structurally similar to α-keto-isopentanoic acid, it was inferred that 3-methylbutanoic acid may be generated from α-keto-isopentanoic acid through a multi-step reaction. 6-Methyl-3,5-heptadiene-2-one and 6-methyl-5-hepten-2-ol were highly correlated with 6-methyl-5-heptene-2-one, geranyl acetone, and *trans*-*β*-ionone, while 6-methyl-3,5-heptadiene-2-ol was highly correlated with 6-methyl-5-heptene-2-one, geranyl acetone, and *trans*-*β*-ionone. 6-methyl-heptadiene-2-one, 6-methyl-5-hepten-2-ol, and 6-methyl-5-heptene-2-one were structurally similar. In the LOX pathway, 1-penten-3-ol is dehydrogenated to produce 1-penten-3-one [47]. Similarly, 6-methyl-5-heptene-2-one might be altered in a single step to produce 6-methyl-3,5-heptadiene-2-one and 6-methyl-5-hepten-2-ol (Figure 5).

## 5. Conclusions

This study used the sensory evaluation and volatilomics analysis of three tomato subgroups’ materials (BIG, *S. lycopersicum*, CER, *S. lycopersicumvar. Cerasiforme*, and PIM, *S. pimpinellifolium*). It was found that 1-Octen-3-ol was an important volatile substance affecting the bitterness of green tomato fruit. Phenylethyl alcohol is an important volatile that affects the sweet and sour taste of red tomato fruit. Branched-chain volatiles are mainly accumulated in PIM and BIG red fruit, and apocarotenoids are mainly accumulated in CER. Branched-chain volatiles and apocarotenoids synergistically regulate the aroma of red tomato fruit. These findings suggest that domestication and improvement have influenced volatile content, and apocarotenoids and branched-chain volatiles synergistically mediated aromatic flavors in red fruits.

## Data Availability

The original contributions presented in the study are included in the article, further inquiries can be directed to the corresponding author.

## Supplementary Materials

The following supporting information can be downloaded at: https://www.mdpi.com/article/10.3390/foods13060879/s1, Figure S1: OPLS DA analysis of quantitative data on volatiles from different subgroups of green and red fruits; Figure S2: Scatter plot of differential volatiles (VIP value greater than 1); Figure S3: Hierarchical cluster analysis of differential volatiles; Figure S4: Correlation analysis of differential volatiles (regions in red and blue indicate positive or negative correlations traits); Table S1: Tomato variety information; Table S2: Tomato fruit sensory evaluation scores; Table S3: Total ion flow chromatogram for tomato during both red and green fruit stages; Table S4: Database of tomato-fruit volatile metabolism; Table S5: Quantitative data on volatiles in immature tomato fruits; Table S6: Quantitative data on volatiles in ripe red tomatoes; Table S7: Correlations between volatile abundance in immature tomato fruits and sensory-evaluation properties; Table S8: Correlations between volatile abundance in mature tomato fruits and sensory-evaluation properties.

## Abbreviations

PCA, principal component analysis; PIM, *S. pimpinellifolium*; CER, *S. lycopersicum var cerasiforme*; BIG, *S. lycopersicum*; OPLS-DA, Orthogonal partial least squares discriminant analysis; NIST, the National Institute of Standards and Technology; FooDB, Food Browse; BCATs, branched-chain amino acid aminotransferase; aKDC, a-ketoacid decarboxylase; AD, Aldehyde dehydrogenases; ZDS, zeta-carotene desaturase; LYC, lycopene beta-cyclase; and CCDs, carotenoid cleavage dioxygenases.
