# Supplementary material for: Volatilomics-Based Discovery of Key Volatiles Affecting Flavor Quality in Tomato
Source: Foods. 2024 Mar 14;13(6):879. doi: 10.3390/foods13060879 (PMC10969657; doi:10.3390/foods13060879)
Supplement: Supplementary file 1 [file foods-13-00879-s001.zip › supplementary Figures.pdf]

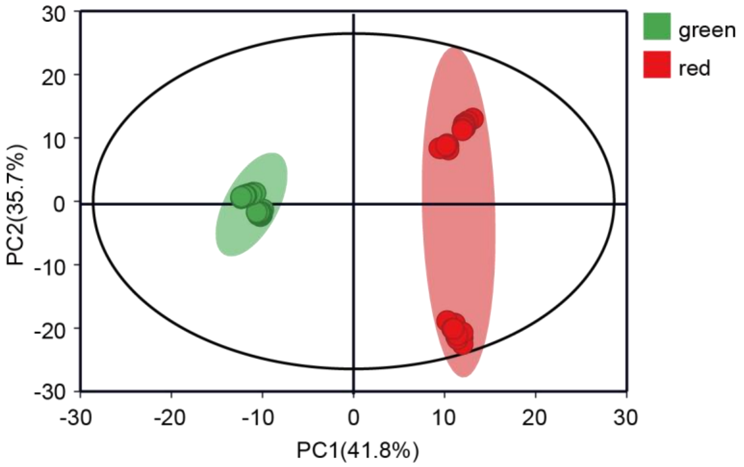

Supplementary Figure S1. OPLS-DA analysis of quantitative data on volatiles from different subgroups of green and red fruits.

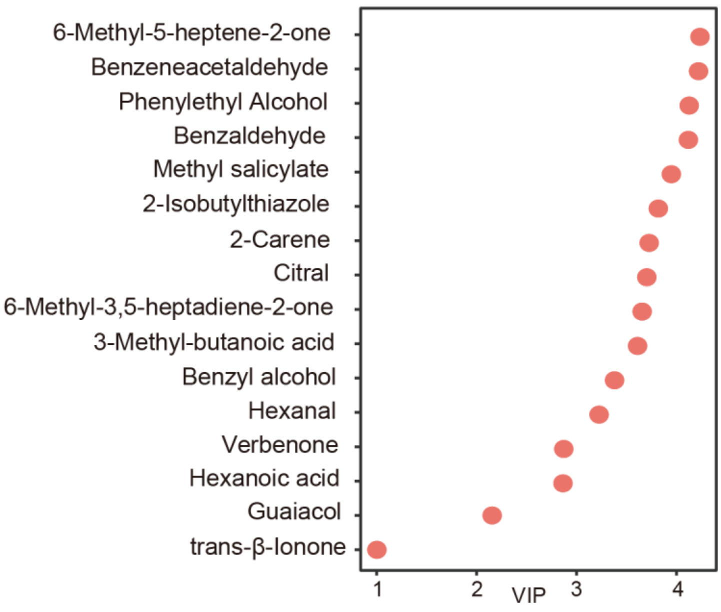

Supplementary Figure S2. Scatter plot of differential volatiles (VIP value greater than 1)

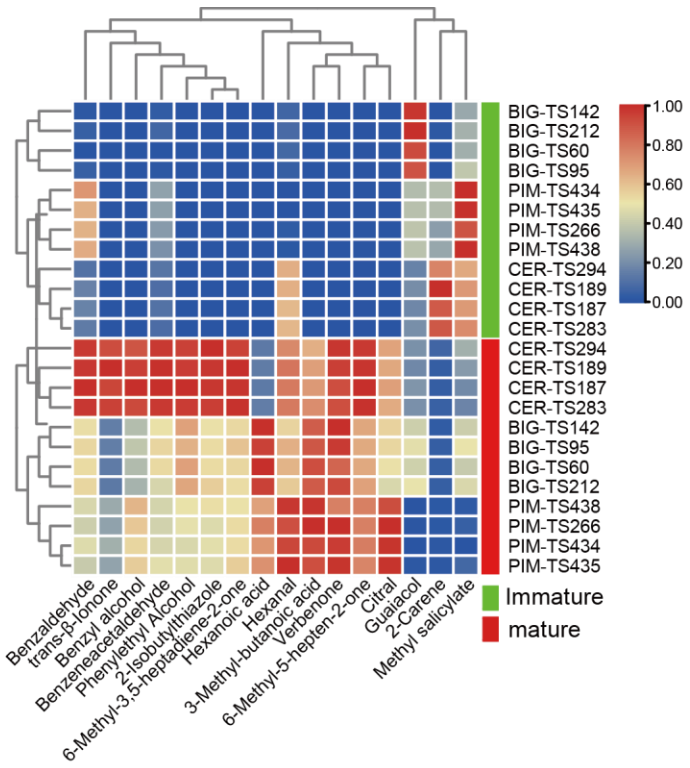

Supplementary Figure S3. Hierarchical cluster analysis of differential volatiles.

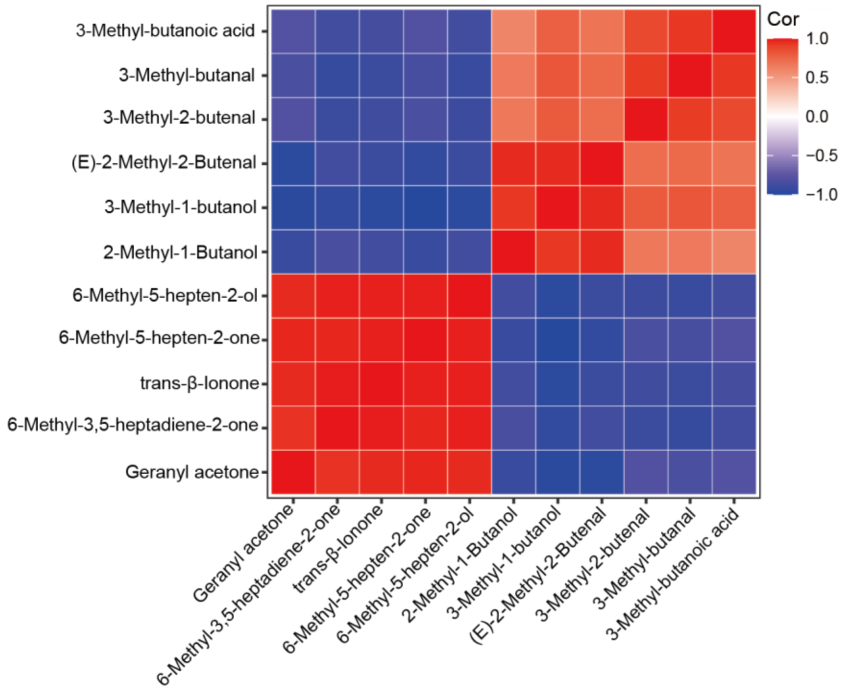

Supplementary Figure S4. Correlation analysis of differential volatiles (Regions in red and blue indicate positive or negative correlations traits).
